# Efficacy of pharmacotherapies for bulimia nervosa: a systematic review and meta-analysis

**DOI:** 10.1186/s40360-023-00713-7

**Published:** 2023-12-02

**Authors:** Sijie Yu, Yuhan Zhang, Chongkai Shen, Fei Shao

**Affiliations:** 1 Center for Rehabilitation Medicine, Department of Psychiatry, Zhejiang Provincial People’s Hospital, Affiliated People’s Hospital, Hangzhou Medical College, Hangzhou, Zhejiang China; 2 The Second Clinical Medical College of Zhejiang, Chinese Medicine University, Hangzhou, Zhejiang China; 3 Hangzhou Xiaoshan No 2 People’s Hospital, Hangzhou, Zhejiang China

**Keywords:** Bulimia nervosa, Drug therapy, Meta-analysis, Antidepressant, Binge-eating

## Abstract

**Objective:**

The main purpose was to evaluate the efficacy and tolerability of different medications used to treat bulimia nervosa (BN).

**Methods:**

Randomized controlled trials (RCTs) were identified from published sources through searches in PubMed, Cochrane Library, Web of Science, and Embase from inception to November 2022. Primary outcomes were changes in the frequency of binge eating episodes and vomiting episodes from baseline to endpoint. Secondary outcomes were differences in the improvement of scores in depressive symptoms, tolerability (dropout due to adverse events) and weight change.

**Results:**

The literature search ultimately included 11 drugs, 33 studies and 6 types of drugs, 8 trials with TCAs (imipramine, desipramine), 14 with SSRIs (fluoxetine, citalopram and fluvoxamine), 6 with MAOIs (phenelzine, moclobemide and brofaromine), 3 with antiepileptic drugs (topiramate), 1 with mood stabilizers (lithium), and 1 with amphetamine-type appetite suppressant (fenfluramine). The reduction in binge eating episodes was more likely due to these drugs than the placebo, and the SMD was -0.4 (95% CI -0.61 ~ -0.19); the changes in the frequency of vomiting episodes (SMD = -0.16, 95% CI -0.3 ~ -0.03); weight (WMD = -3.05, 95% CI -5.97 ~ -0.13); and depressive symptoms (SMD = -0.32, 95% CI -0.51 ~ -0.13). However, no significant difference was found in dropout due to adverse events (RR = 1.66, 95% CI 1.14 ~ 2.41).

**Conclusions:**

This meta-analysis indicates that most pharmacotherapies decreased the frequency of binge-eating and vomiting episodes, body weight, and depressive symptoms in BN patients, but the efficacy was not significant. In each drug the efficacy is different, treating different aspects, different symptoms to improve the clinical performance of bulimia nervosa.

**Supplementary Information:**

The online version contains supplementary material available at 10.1186/s40360-023-00713-7.

## Background

Bulimia nervosa (BN) refers to recurrent episodes of overeating in which a larger amount of food is consumed than individuals would consume at similar times and on similar occasions, during which they feel unable to control their eating; recurrent inappropriate compensatory behaviors to prevent weight gain, such as self-induced vomiting, abuse of laxatives, diuretics or other medications, fasting, or excessive exercise, and self-critical of body size and weight; binge eating is accompanied by inappropriate compensatory behavior, at least once a week on average over 3 months, according to Diagnostic and Statistical Manual of Mental Disorders (DSM)-5 criteria [1].

Eating disorders are believed to be multifactorial, with genetic predisposition, environmental factors, and psychological characteristics involved. The lifetime prevalence of BN is between 0.9% and 3%, and the 12-month prevalence is 0.4%, with the average age of onset being 16 to 17 years old [2]. Approximately 3% of females and more than 1% of males suffer from BN. Researchers have mainly observed that BN affects young, western females, but it has also been reported in males and females worldwide [3].

Binge eating is typically triggered by dysphoric mood stages and is usually accompanied by depression and self-criticism. There is a strong association between eating disorders and depression according to a broad review of the literature. A recent study reported that the most common comorbidities among people with eating disorders were mood disorders (43%) and anxiety disorders (53%) [4]. Approximately 80–90% of BN patients were reported to have had at least one episode of a mood disorder in their lifetime, mostly a depressive episode [5]. Despite the high comorbidity between eating disorders and depression, it is unclear whether depression antedates, coexists with, or is a result of eating disorders [6].

The treatment of BN includes nutritional therapy, somatic therapy, psychoactive medication, psychotherapy, and psychotherapy combined with medication. Generally, patients with BN benefit from short-term psychotherapy, such as cognitive behavioral therapy (CBT) [7]. There are some network meta-analyses of BN treatment that found that psychotherapy, particularly CBT, is the best treatment for BN [8, 9]. It has been suggested that interpersonal psychotherapy (IPT) can produce similar results to CBT, but it takes longer to achieve these results [10, 11].

Additionally, antidepressant use has been shown to benefit BN patients, and multidisciplinary, team-based therapy is the most successful [7]. It appears that all types of antidepressants seem to be beneficial to some degree in reducing bulimic symptoms in many patients [12]. However, there is no conclusive proof that one type of drug is more beneficial than another. Topiramate may be helpful for the short-term treatment of BN because it can reduce body weight and binge eating frequency, according to a systematic evaluation [13].

In previous studies, separate papers on topiramate and antidepressants have been published. No research covered all medications in a comprehensive way. Some articles did not have subgroups according to the length of therapy, and some had no classification according to the type of drugs. To fill this gap, we performed a systematic review and meta-analysis of double-blind, randomized controlled trials (RCTs) for all drugs. In addition, the subgroups were separated based on the length of the therapy and the type of drugs used. We do not yet know which parts of the various medications are more effective or how well they are tolerated. The existing research evidence is mixed. Therefore, the purpose of this article is to show the efficacy and tolerability of various medications for bulimia nervosa.

## Materials and methods

### Search strategy

**Fig. 1:**
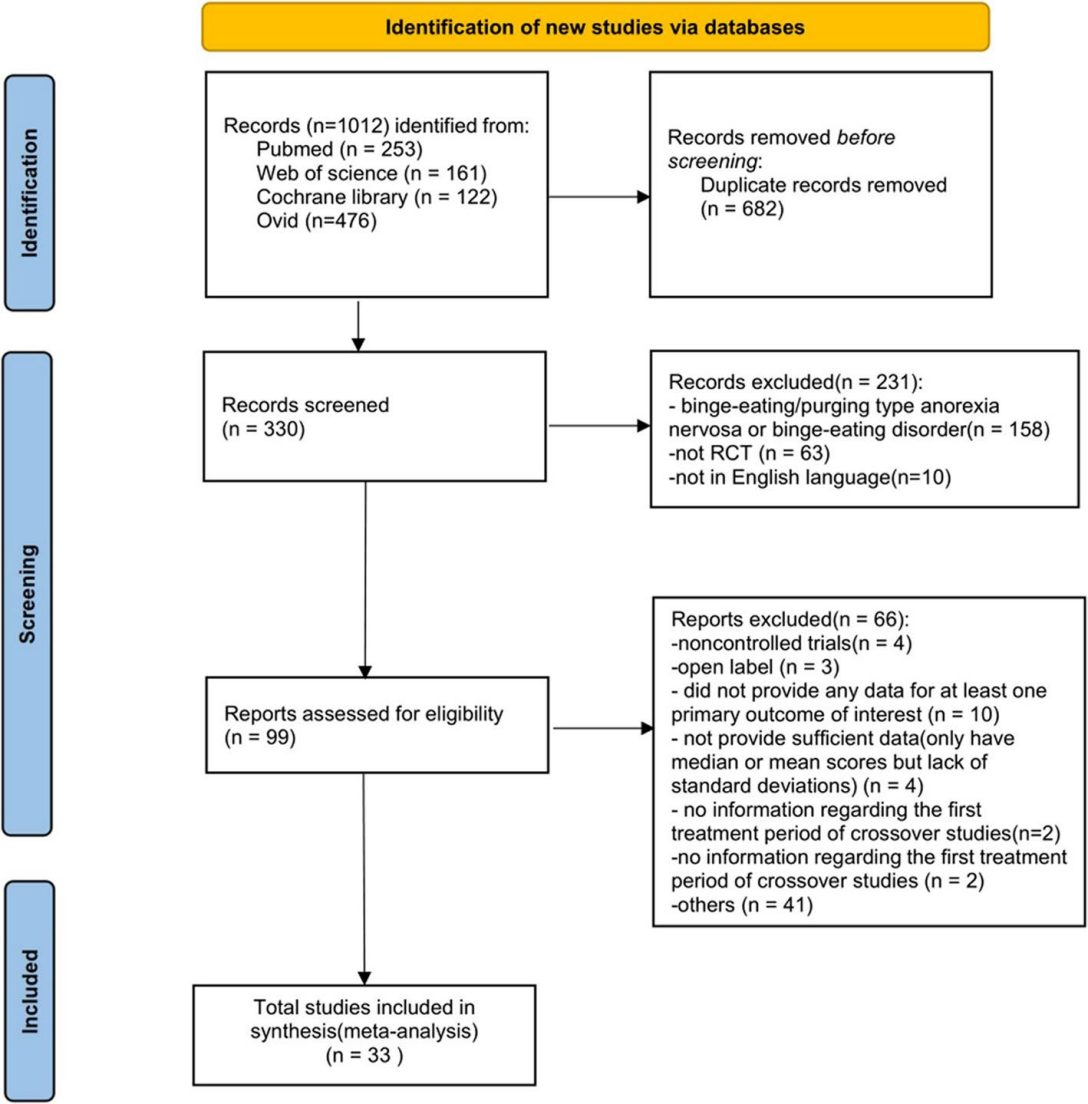
Flow diagram of study selection: article search strategy results

A comprehensive literature search was conducted according to the preferred reporting items of the systematic review and meta-analysis (PRISMA) guidelines [14]. A primary search was conducted using PubMed, Cochrane Library, Web of Science, Embase and studies published from the first RCT until October 2023. The search terms used were “bulimia nervosa” and “treatment with psychotropics or pharmacotherapy or antidepressants or tricyclic* or SSRIs or SNRIs or MAOI or topiramate or anticonvulsants or psychostimulants or stimulants or medicine or medications or drugs or drug therapies”. Studies were restricted to the English language. All retrieved studies were entered into the reference manager software. Duplicates were removed, and the titles and abstracts of the remaining studies were independently assessed for eligibility by three authors (YSJ, ZYH and SCK). Following this assessment, the full texts of all potentially eligible studies were examined for inclusion in the review. The selection process was overseen by the senior author (SF), who resolved any potential disagreements. The selection process was documented using the PRISMA flow diagram (Fig. 1). This review was registered in the International Prospective Register of Systematic Reviews (PROSPERO), registration number CRD42022380430.

### Eligibility criteria

The inclusion criteria were pharmacotherapies with a diagnosis of BN according to either the DSM-III, III-R, IV, V or the International Classification of Diseases (ICD)-10. The search was limited to humans. Language was limited to English. We excluded people with binge-eating/purging type anorexia nervosa or binge-eating disorder (BED) as defined in DSM-V. If no information regarding the first treatment period of crossover studies could be obtained [15, 16], they would be excluded. Only RCTs were included, and most excluded studies were reviews, case reports, letters, open label studies [17–19], and noncontrolled trials [20–23]. Some studies did not provide any data for at least one primary outcome of interest [24–33] or did not provide sufficient data (only have median or mean scores but lack standard deviations) [34–37], so these trials could not be included in the analysis. Wood (1993) [38] followed up with FBNCSG (1992) [39]; Dalai et al. (2017) [40] and Safer et al. (2020) reported on the same trial [41]; Mitchell et al. (1984) [42] was not randomized (with a high discontinuation rate). Therefore, they were excluded. Some of these articles covered pharmacotherapy and psychotherapy, and we compared psychotherapy plus pharmacotherapy with psychotherapy alone to compare the effects of medication. Data for at least one primary outcome of interest will be reported.

### Data extraction

Information from eligible studies was extracted and recorded in an electronic spreadsheet designed by the authors. The following information was extracted: a. Authors—Year of publication; b. Treatment—drug, dosage and study duration; c. Participants—Age, number of participants in the drug and placebo groups; and d. Outcomes. One member of the research team abstracted relevant data from each included article. A senior member of the research team reviewed each abstraction for accuracy and completeness. The key characteristics of all included trials are summarized in Table 1.

**Table 1:** key characteristics and Cochrane quality risk assessment of all included trial

study	treatment	outcome	dose (mg/d)	Duration (weeks)	number of patients		Age, years (SD)		random	allocation concealment	double blindness	outcome data integrity	selective reporting	other bias
					drug	placebo	drug	placebo						
Attia 1998 [43]	fluoxetine	weight, BDI, drop outs due to adverse events	60	7	15	16	29.1 (7.2)	23.4 (6.4)	L	U	L	L	L	L
Goldstein 1995 [44]	fluoxetine	frequency of vomiting and binge eating episodes, HAMD, drop outs due to adverse events	60	16	296	102	27 (17.6)	26 (17.6)	L	U	L	L	L	L
Goldbloom 1997 [45]	fluoxetine, CBT	frequency of vomiting and binge eating episodes, BDI	60	16	29	23	25.8 (5.5)	25.8 (5.5)	L	U	H	L	L	L
Grilo 2005 [46]	fluoxetine	frequency of binge eating episodes, BDI, drop outs due to adverse events	60	16	27	27	44.3 (9.5)	43.6 (8.5)	L	U	L	L	L	L
Walsh 2000 [47]	fluoxetine	frequency of binge eating and purging episodes, BDI	60	NA	13	9	32.0 (7.8)	27.8 (5.2)	L	U	L	U	L	L
Romano 2002 [48]	fluoxetine	frequency of vomiting and binge eating episodes, drop outs due to adverse events	60	8	76	74	29.5 (7.0)	30.0 (9.3)	L	U	L	L	L	L
Fichter 1991 [49]	fluoxetine	HAMD, weight	60	7	20	20	26.5 (NA)	24.6 (NA)	L	U	L	L	L	L
FBNC 1992 [39]	fluoxetine	frequency of binge-eating and purging episodes, weight, HAMD, drop outs due to adverse events	20,60	8	129	129	26.4 (6.2)	27.7 (8.0)	L	U	L	L	L	L
Beumont 1997 [50]	fluoxetine	frequency of vomiting and binge eating episodes, HAMD	60	8	34	33	24.2 (4.5)	25.1 (5.8)	L	U	L	L	L	L
Jacobi 2002 [51]	fluoxetine, CBT	frequency of bing eaing and purging episode, BDI	20–60	16	18	19	26.0 (5.8)	26.0 (5.8)	L	U	H	L	L	L
Kanerva 1994 [52]	fluoxetine	weight, HAMD, drop outs due to adverse events	60	8	24	26	25.2 (9.9)	25.2 (9.9)	L	U	L	U	L	L
Marcus 1990 [53]	fluoxetine	weight, BDI	60	52	18	15	40.3 (9.5)	40.9 (7.9)	L	L	L	L	L	L
Sundblad 2005 [54]	citalopram	frequency of binge eating episodes	20–40	12	18	14	26.0 (NA)	28.0 (NA)	L	U	L	L	L	L
Fichter 1997 [28]	fluvoxamine	drop-outs due to adverse events, CGI,HAMD	100–300	15	37	35	25.2 (NA)	23.7 (NA)	L	L	L	L	L	L
Safer 2020 [41]	topiramate	frequency of binge eating episodes, drop outs due to adverse events	3.75 /23; 15 /92	12	22	22	42.9 (10.1)	42.9 (10.1)	L	U	L	L	L	L
Nickel 2005 [55]	topiramate	frequency of bing eating episodes, weight	25–250	10	30	30	21.5 (3.1)	21.5 (3.1)	L	L	L	L	L	L
Hoopes 2003 [56]	topiramate	frequency of binge and purge days, drop outs due to adverse events	25–400	10	35	34	29.0 (9.7)	29.6 (8.1)	L	L	L	L	L	L
Fahy 1993 [57]	fenfluramine	weight, frequency of binge eating and vomiting episodes	45	8	20	23	23.0 (0.6)	25.0 (1.4)	L	U	L	L	L	L
Carruba 2001 [58]	moclobemide	frequency of binge eating and vomiting episodes, HAMD, drop outs due to adverse events	600	6	38	39	25.6 (0.8)	25.1 (0.9)	L	U	L	L	L	L
Pope 1983 [59]	lmipramine	frequency of binge eating episodes, HAMD, dropout due to adverse events	50	6	11	11	27.9 (6.2)	27.6 (6.3)	L	U	L	L	L	L
Alger 1991 [60]	lmipramine	weight, BDI, dropout due to adverse events	50–150	8	12	11	40 (0.6)	30 (0.8)	L	U	L	L	L	L
Agras 1987 [61]	lmipramine	frequency of binge eating and purging episodes, BDI	50–300	16	10	10	30.3 (NA)	31.5 (NA)	L	U	L	L	L	L
Rothschild 1994 [62]	lmipramine	HAMD	150	6	6	10	32.2 (47.2)	29.7 (24.7)	L	U	L	U	L	L
McCann 1990 [63]	desipramine	frequency of binge eating episodes, BDI, weight	25–300	12	15	15	NA	NA	L	U	L	L	L	L
Agras 1992 [64]	desipramine, CBT	frequency of binge eating and purging episodes	50–350	24	12	23	29.6 (8.9)	29.6 (8.9)	L	U	L	L	L	L
Walsh 1997 [65]	desipramine, CBT,SPT	frequency of binge eating and vomiting episodes, BDI, weight	200–300	16	23	25	26.1 (5.7)	25.8 (4.4)	L	U	L	L	L	L
Walsh 1991 [66]	desipramine	HAMD, BDI, dropout due to adverse events	200–300	6	40	38	25.7 (5.6)	24.8 (4.5)	L	U	L	L	L	L
Walsh 1984 [67]	phenelzine	frequency of binge eating episodes, HAMD, drop outs due to adverse events	60–90	8	9	11	26.9 (5.1)	26.0 (4.5)	L	U	L	L	L	L
Walsh 1988 [68]	phenelzine	the frequency of binge eating episodes, BDI, HAMD, drop outs due to adverse events	60–90	8	31	31	26.9 (4.3)	27.1 (5.2)	L	U	L	L	L	L
Walsh 1985 [69]	phenelzine	the frequency of binge eating episodes, HAMD, drop outs due to adverse events	60–90	8	14	16	27.8 (4.7)	27.2 (5.3)	L	U	L	L	L	L
Rothschild 1994 [62]	phenelzine	HAMD	45	6	8	10	37.1 (27.5)	29.7 (24.7)	L	U	L	U	L	L
Hsu 1991 [70]	lithium	the frequency of binge eating and vomiting episodes, HAMD, BDI, weight	300	8	27	23	25.4 (7.0)	25.4 (7.0)	L	U	L	L	L	L
Kennedy 1993 [71]	brofaromine	the frequency of vomiting and binge eating episodes, HAMD, drop outs due to adverse events	175	8	19	17	27.6 (6.7)	25.9 (6.4)	L	U	L	L	L	L

*BDI* Beck Depression Inventory, *HAMD* Hamilton Depression Rating Scale, *L* low risk, *U* Unclear risk, *H* High risk

### Outcome measurement

The primary outcomes of interest were changes in the frequency of binge eating episodes and changes in the frequency of vomiting episodes from baseline to endpoint. Patients recorded binge and vomiting episodes (i.e., purge, laxative or diuretic use, and days of fasting), as well as the time and quantity of medication taken, in daily diaries to assist in accurate reporting. Secondary outcomes were differences in the improvement of scores in depressive symptoms from baseline to endpoint, including the Hamilton Depression Rating Scale (HAMD) and Beck Depression Inventory (BDI); tolerability of treatment, the number of patients dropping out during the study due to adverse events; and weight change from baseline to endpoint.

### Quality assessment

Two researchers independently completed these RCTs according to the Cochrane Collaboration’s tool for assessing the risk of bias [72]. In the case of disagreement, a third researcher participated in the discussion to determine the overall literature quality. The evaluation metrics included random sequence generation, assignment hiding, double blindness, outcome data integrity, selective reporting of study results, and other sources of bias. According to these indicators, the included literature was evaluated as “high risk,” “low risk,” and “unknown.”

### Statistical analysis

Data were analyzed using Review Manager (RevMan) 5. The risk ratio (RR) with 95% confidence intervals (CI) was calculated for dichotomous outcome (drop-outs) and the standardized mean difference (SMD) of continuous outcomes (changes in the frequency of binge eating and vomiting episodes, the scores of depressive symptoms), while weighted mean difference (WMD) was calculated for weight. A random effects model was used to estimate RR and SMD since it takes into account any differences between studies, even if there is no statistically significant heterogeneity between them [73]. Heterogeneity was examined using I^2^ (25%, 50%, and 75% for low, medium, and high heterogeneity, respectively) [74]. For the primary outcomes, subgroup analyses were performed for the duration of treatment (up to 10 weeks of treatment and 10 or more weeks of treatment) and class of drugs.

## Results

### Description of studies

In this meta-analysis, 1012 references were obtained through preliminary database inspection. The study selection process is shown in Fig. 1. The literature search ultimately included 11 drugs and 33 studies. These trials were used for at least one of the main comparisons. Studies included 6 types of drugs: 1. selective serotonin reuptake inhibitors (SSRIs): 10 studies compared fluoxetine with placebo: [39, 43, 44, 46–50, 52, 53]; only two studies compared CBT plus fluoxetine with CBT to show the efficacy of fluoxetine [45, 51] and citalopram versus placebo [54] and fluvoxamine [28]; 2. tricyclic antidepressants (TCA): lmipramine [59–62] and desipramine vs. placebo [63, 66]; 2 studies compared CBT plus desipramine with CBT [64, 65]; 3. monoamine oxidase inhibitors (MAOI): phenelzine [62, 67–69], moclobemide [58], and brofaromine [71]; 4. Antiepileptic drugs: topiramate [41, 55, 56]; 5. mood stabilizer: lithium [70]; 6. methamphetamine-type appetite suppressant: fenfluramine [57]. When different articles were found for the same trial, only the article with the desired outcome was selected.

FBNCSG 1992 [39] compared 20 and 60 mg of fluoxetine to placebo, and we considered only the 60 mg group for comparisons. This trial found that a daily dose of 60 mg was more effective than a daily dose of 20 mg of antidepressants. If there were other intervention groups in the study, we only included the two groups of needed drugs and placebo, and the other groups were not considered, such as Grilo et al. 2005 [46] and Sundblad et al. 2005 [54]. Rothschild et al. 1994 [62] divided the treatment into three groups of imipramine, phenelzine and placebo, and we analyzed the comparison between the two drugs and placebo.

Patients were mostly adult and young adult females; few adolescents and males were included. Four continuous outcomes were used in this review. Two concerned changes in bulimic symptoms and were considered primary efficacy outcomes: changes in the frequency of binge eating episodes and changes in the frequency of vomiting episodes from baseline to endpoint. The other continuous outcomes were the improvement of scores in depressive symptoms and weight change. When analyzing improvement in depressive symptoms, studies were pooled, even if the depression rating scale was different, as long as all trials were comparable. The dichotomous outcomes used concerned tolerability of treatment, which means the number of drop-outs due to adverse events. The outcomes recorded where adverse experiences were so severe that patients stopped treatment prematurely. This was extracted from 19 trials.

### Risk of bias assessment

All included trials were assessed for risk of bias using the Cochrane Collaboration tool, and details are provided in Table 1. All the studies were randomized. For allocation concealment, only four studies [28, 53, 55, 56] were judged at low risk. For example, envelopes containing individual randomization information were available at each site to be opened in cases of medical necessity; tablets were supplied in numbered boxes, and both subjects and clinicians were blinded regarding medicine assignment. Other studies did not describe the hidden methods sufficiently to make a definitive judgment, so they were unclear. Most of the research was double-blind. Two studies [45, 51] did not use a blinded protocol, so they were judged as high risk. For outcome data integrity, only three studies [47, 52, 62] were of unclear risk because they did not report the number or reasons for dropout. All studies had a low risk of selective reporting and no other bias. Overall, only two studies were at high risk, and most studies were at unclear risk, so the literature quality was generally good.

### Effects of treatment

#### Changes in the frequency of binge eating episodes

This outcome was reported in 23 trials (SSRIs: fluoxetine [44–46, 48–51], citalopram [54]; MAOI: moclobemide [58], brofaromine [71]; mood stabilizer: lithium [70]; TCA: lmipramine [59, 61], desipramine [63–66]; antiepileptic drugs: topiramate [41, 55]; amphetamine-type appetite suppressant: fenfluramine [57]), including 1376 participants.

**Fig. 2:**
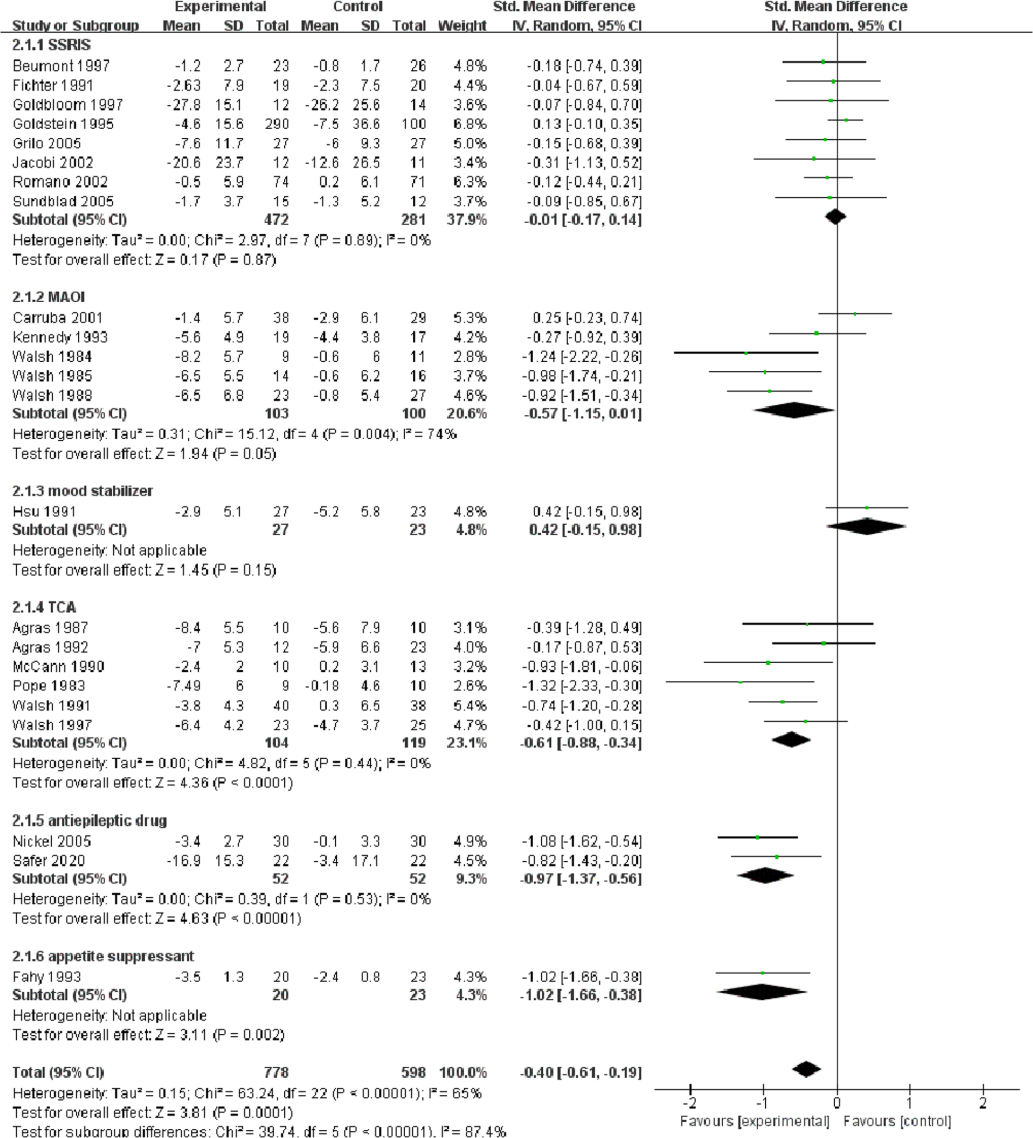
Forest plot of binge-eating episodes

SSRIs (eight trials, 472 treated with drugs and 281 with placebo) showed an SMD of -0.01 (95% CI -0.17 ~ 0.14), with no heterogeneity. If we removed two studies [45, 51] that included CBT, the SMD would be 0 (95% CI -0.16 ~ 0.16). Neither was statistically significant. Concerning MAOIs (five trials, 103 patients in the drug group and 100 patients in the placebo group), the SMD was -0.57 (95% CI -1.15 ~ 0.01), and the heterogeneity was high (I^2^ = 74). When Carruba et al. (2001) [58], which was for moclobemide, was excluded, the heterogeneity declined to 18%. TCAs (six studies, 223 patients) showed an SMD of -0.61 (95% CI -0.88 ~ -0.34). If we eliminated those two studies with CBT [64, 65], the SMD would be -0.79 (95% CI -1.13 ~ -0.44). Regarding the antiepileptic drug topiramate (two studies, 52 patients per group), the SMD was -0.97 (95% CI -1.37 ~ -0.56), with no heterogeneity. The SMD in appetite suppressant was -1.02 (95% CI -1.66 ~ -0.38). For mood stabilizer-lithium, the SMD was 0.42 (95% CI -0.15 ~ 0.98), proving that it was not effective in bulimia nervosa. For all drugs, the SMD was -0.4 (95% CI -0.61 ~ -0.19), with medium heterogeneity (I^2^ = 65), indicating that a short-term reduction in binge eating episodes was more likely for these drugs than for placebo (Fig. 2). There was little effect on validity and heterogeneity in the studies with or without psychotherapy plus medication [45, 51, 64, 65].

#### Changes in the frequency of vomiting episodes

This outcome included eleven trials (SSRIs: fluoxetine [44, 45, 48, 50, 51]; MAOI: moclobemide [58], brofaromine [71]; TCA: desipramine [65, 66]; mood stabilizer [70]; amphetamine-type appetite suppressant: fenfluramine [57]), with 578 participants in the experimental group and 387 in the control group.

**Fig. 3:**
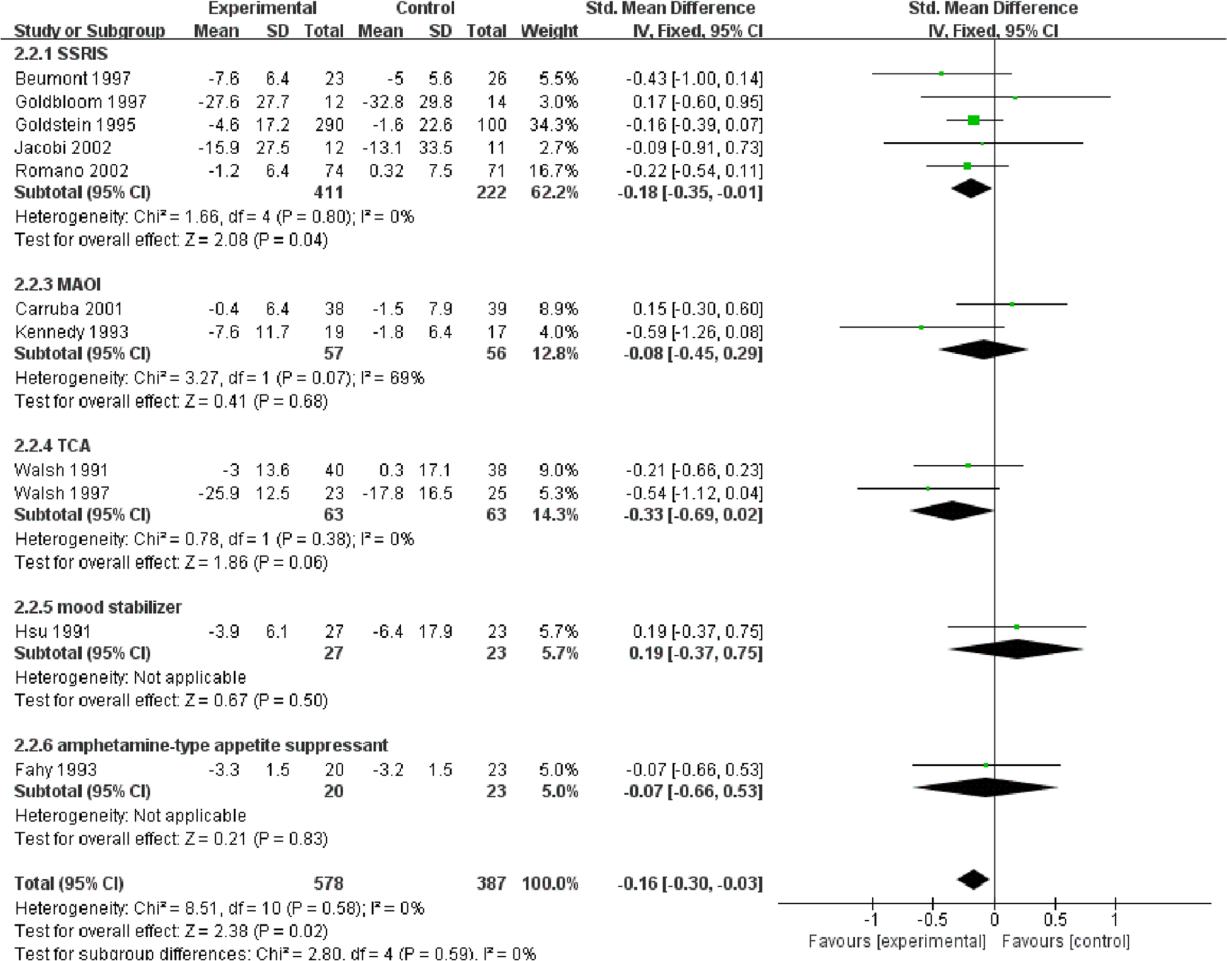
Forest plot of vomiting episodes

SSRIs, including only fluoxetine (five trials, 411 treated with drugs and 222 with placebo), showed an SMD of -0.18 (95% CI -0.35 ~ -0.01), with no heterogeneity. When two studies with CBT were removed [45, 51], the SMD was -0.2 (95% CI -0.38 ~ -0.03), which shows little difference from before. However, MAOI (two trials) indicated that the SMD was -0.18 (95% CI -0.9 ~ -0.55), and the heterogeneity was medium (I^2^ = 69). Concerning TCA (two trials, 63 patients per group), the SMD was -0.33 (95% CI -0.69 ~ 0.02), and there was no heterogeneity, indicating no statistical significance. When excluding the study with CBT [65], the SMD was -0.21 (95% CI -0.66 ~ -0.23). Regarding mood stabilizers (only one trial), the SMD was 0.19 (95% CI -0.37 ~ -0.75), indicating that they were not effective. For amphetamine-type appetite suppressant [57], the SMD was -0.07 (95% CI -0.66 ~ 0.53), which was also not statistically significant. For all drugs, the SMD was -0.16 (95% CI -0.3 ~ -0.03), and no heterogeneity in the results of these 11 trials was found (Fig. 3). The exclusion of several studies in which drugs were combined with CBT also did not greatly affect the results.

### Weight

For fluoxetine (SSRI; five trials [39, 43, 49, 52, 53]), the WMD was -3.57 (95% CI -6.73 ~ -0.41), with heterogeneity (I^2^ = 57). When removing Marcus et al. (1990) [53], who searched for obese binge-eaters and lasted for 52 weeks, heterogeneity was eliminated. TCA [60, 63, 65] showed a WMD of -2.73 (95% CI -6.38 ~ 0.92), which meant no statistical significance. Concerning amphetamine-type appetite suppressants [57], WMD was 4.0 (95% CI 2.89 ~ 5.11). This meant that fenfluramine could not cause weight loss. Topiramate [41, 55] showed remarkable efficacy in weight loss in bulimic patients, with a WMD of -5.24 (95% CI -7.63 ~ -2.86). The WMD of all drugs was -3.05 (95% CI -5.97 ~ -0.13), and the heterogeneity was very high (I^2^ = 92). Therefore, we excluded the amphetamine-type appetite suppressant for sensitivity analysis; the heterogeneity declined to 66%, and the WMD was -3.87 (95% CI -5.87 ~ -1.87) (Supplementary Fig. S1).

### The depression scores

We used HAMD and BDI scale scores to assess depressive symptoms. SSRIs, including 10 trials [43–52], showed an SMD of -0.07 (95% CI -0.22 ~ 0.08), with no heterogeneity. When removing the two studies with CBT [45, 51], the result did not change much. Concerning MAOIs (six trials [58, 62, 67–69, 71], 111 participants in the experimental group and 120 in the control group), the SMD was -0.50 (95% CI -0.95 ~ -0.06), with 60% heterogeneity. If Carruba et al. (2001) [58] were excluded, the heterogeneity declined to 3%, indicating that the study was research on moclobemide. For TCA, there were seven trials [59, 61–63, 65, 66], and the SMD was -0.52 (95% CI -1.00 ~ -0.03). The SMD was -0.32 (95% CI -0.51 ~ -0.13) in total drugs, and the heterogeneity was 53% (Supplementary Fig. S2).

### Dropouts due to adverse events

For SSRIs (7 trials [28, 39, 43, 44, 46, 48, 52]), the RR was 1.68 (95% CI 1.11 ~ 2.54). MAOI (5 trials) [58, 67–69, 71] showed an RR of 2.24 (95% CI 0.63 ~ 7.91); the RR in TCA (3 trials) [59, 60, 66] was 2 (95% CI 0.32 ~ 12.5). For the antiepileptic drug topiramate [41, 56], the RR was 1.35 (95% CI 0.27 ~ 6.75). The total RR was 1.66 (95% CI 1.14 ~ 2.41), with 8% heterogeneity (Supplementary Fig. S3). There was no statistical significance in individuals who dropped out due to adverse events. However, overall, more participants dropped out because of the drug than the placebo.

### 10-week duration of treatment

Ten trials [41, 44–46, 51, 54, 61, 63–65] reported binge eating episodes had a duration longer than 10 weeks, and thirteen trials [48–50, 55, 57–59, 66–71] lasted less than 10 weeks. No significant difference was found between SMD and 95% confidence intervals for these two groups in binge eating episodes【SMD = -0.21 (-0.45 ~ 0.04) versus -0.5 (-0.8 ~ 0.19)】. However, the heterogeneity in < 10 weeks was high (I^2^ = 72) (Supplementary Fig. S4). Four trials reported vomiting episodes with a duration of longer than 10 weeks, and seven trials had a duration of up to 10 weeks. No significant difference or statistical significance was found between SMD and 95% confidence intervals for these two groups in vomiting episodes 【SMD = -0.18 (-0.38 ~ 0.02) versus -0.15 (-0.33 ~ 0.03)】, with no heterogeneity (Supplementary Fig. S5).

## Discussion

This meta-analysis researched all drugs for bulimia, including the newer antiepileptic topiramate and several older drugs in addition to antidepressants, and investigated their efficacy on the frequency of binge eating and vomiting episodes, weight loss, improvement of depressive symptoms, and the adverse events dropout rate. Twenty-eight RCTs (placebo-controlled) were identified with medication alone as the primary intervention, and only 4 were identified with medication associated with or in combination with a psychotherapy intervention.

Overall, we found that, compared to the control group, TCA, topiramate and fenfluramine were associated with a reduction in the frequency of binge eating episodes per week. In addition, topiramate was also effective in lowering body weight in bulimic patients. SSRIs (fluoxetine) were associated with a reduction in the frequency of vomiting episodes per week and induced a greater weight reduction than the control group. MAOI and TCA slightly improved depression symptoms. The mood stabilizer lithium was ineffective against binge eating and vomiting. Relatively speaking, the tolerability of SSRIs and topiramate was relatively good.

SSRIs induced greater weight loss than placebo. However, we did not know if weight loss with SSRIs was related to a reduction in the frequency of vomiting and/or decreased appetite or to metabolic effects, as these parameters were not assessed in these studies. The better tolerability of SSRIs may be related to their short-term effect on body weight [6]. Although no significant difference in fluoxetine depression scores was observed in the results, the results were not unexpected, as in some trials, both treatment groups had scores in the non-depression range at baseline [44, 48]. Interestingly, after starting treatment, placebo-treated patients reported more depression than fluoxetine patients, suggesting that fluoxetine may have a mood-stabilizing effect on bulimia patients [44]. The efficacy of fluoxetine was independent of whether it was associated with depression [39], and its effect may be mediated by changes in brain serotonin activity, abnormalities of which have been documented in BN [75, 76]. Fluoxetine was the most commonly used antidepressant for BN. Although it was not statistically significant in reducing the frequency of binge eating, its reduced frequency of vomiting episodes, weight loss and better acceptability may justify its use as a first-line antidepressant in BN. FBNCSG 1992 [39] found that a daily dose of 60 mg was more effective than a daily dose of 20 mg of antidepressants. The most common adverse events were insomnia, nausea, asthenia, and anxiety.

The emotional stabilizer topiramate was useful in the treatment of BN because it decreased the frequency of binge eating episodes and resulted in significant weight loss. The synergistic effect of topiramate on weight loss among patients is worth further study, especially as weight loss is a significant challenge in comorbid BED and obesity [77]. Topiramate has several mechanisms of action: blocking glutamate neurotransmission, increasing GABA activity, and inhibiting voltage-gated calcium and sodium channels. Its efficacy in treating eating disorders was considered effective due to its inhibitory effect on kainate/AMPA glutamate receptors [78]. The most common side effects of topiramate were dry mouth, somnolence, paresthesia/tingling, dysgeusia, and anxiety. The limited number of studies with small sample sizes makes it difficult to judge the size of the actual effect.

Although through this meta-analysis, we found that MAOI and TCA could reduce binge eating and depression symptoms in BN patients, they were not commonly used clinically due to their high adverse events and poor tolerability. In combination with many drugs, MAOIs can cause serious side effects, such as increased blood pressure, gastrointestinal discomfort, dizziness, insomnia, muscle weakness, blurred vision, and difficulty breathing. The most common side effects of TCA were anticholinergic adverse events, central nervous system toxic events, and cardiovascular toxic events.

Fenfluramine, an amphetamine-type appetite suppressant, could reduce binge eating episodes in BN but was not an effective treatment for the severe abnormal eating disorder of BN. In addition, given the drug's lack of antidepressant effects, it did not lead to an emotion-dependent improvement in abnormal eating behaviors [57]. The additional support of a hospital environment may be needed for the drug to be effective, and plasma fenfluramine levels may have fallen below a therapeutic range after several hours. The most common effects reported were drowsiness, headache, and unsteadiness. The mechanism of the drug in suppressing binge eating in BN patients has not been clarified [79].

For this article, the patients with BN enrolled in the trial were generally similar in terms of duration of disease, settings, age, and symptom severity. Most of the studies included patients with strictly defined bulimia nervosa, according to the diagnostic criteria used in the studies. Most patients did not develop severe depression or other serious complications. The dropout rate observed in trials evaluating drug treatment can be due to adverse events, lack of efficacy, and other factors. Antidepressants may be an effective component of initial treatment options for patients with BN. They may be particularly beneficial in treating patients with significant comorbid symptoms such as anxiety, depression, and compulsion or in patients who have previously failed psychosocial therapies. In general, when compared to placebo, a single antidepressant medicine was clinically effective for the treatment of BN, but the effect was modest.

Compared with the previous meta-analysis, one discussed the effects of antidepressants and placebo controls on BN and excluded the studies on psychotherapy [80], another discussed only topiramate monotherapy treating BN and BED [13], and few sample sizes and trials were included. In Svaldi et al., the main comparison was between medication and psychotherapy, and the efficacy of each drug was not detailed [9]. Compared with Fornaro et al., we added outcome indicators of body weight and dropouts due to adverse events in this paper and divided them into subgroups according to the length of time and types of drugs [81]. Therefore, we now discuss the efficacy of all drugs used to treat BN and add a few more trials to my study for each type of drug.

There were some limitations in our study that should be reported. Because the number of articles for several drugs was insufficient, no network meta-analysis was performed to compare which drug was more effective. Most of the literature is very old and lacks proper methodology, but no new experiments have been published, which may give a hysteretic result. In addition, we encountered difficulties in obtaining some data. Some articles did not contain data with standard deviation. Better access to all data may have facilitated and enhanced the implementation of this meta-analysis. There was considerable heterogeneity in the outcome of the frequency of binge-eating episodes and body weight. Despite the random-effects model and sensitivity analysis, only one type of drug heterogeneity was reduced, not the overall heterogeneity. We were unable to account for these differences. In addition, it remains uncertain whether these benefits assessed in short-term trials translate into long-term health outcomes. There were only a small amount of data to allow evaluation of longer-term effects or durability of pharmacotherapy-only therapy for BN.

It is worth noting that the number of studies and trials declined over time, with few new studies in recent years. This may be because clinicians and patients find psychotherapy more convenient, more effective, and more acceptable. In some studies, CBT combined with medication, both CBT plus medication and CBT alone have been found to be superior to medication alone in reducing binge eating and vomiting [48, 64, 65]. Continuing CBT appeared to prevent relapse for up to 72 weeks in patients who stopped their medication [48]. Future studies should systematically include bulimia patients with concomitant anorexia nervosa, major depression, anxiety disorder, personality disorder, obsessive–compulsive disorder, and other related clinical symptoms and evaluate their impact on prognosis to improve the universality of the results. In addition to fluoxetine, the effects of SSRIs and newer antidepressants still need to be studied. We should also explore which drug is more effective for bulimia.

## Conclusion

In summary, this meta-analysis indicates that most pharmacotherapies decreased the frequency of binge-eating and vomiting episodes, body weight, and depressive symptoms in BN patients, but the efficacy was not significant. In each drug, the efficacy is different, treating different aspects, different symptoms to improve the clinical performance of BN patients. This provides guidance to clinicians on the direction of drug use in BN patients. Pharmacotherapy has the potential to improve compliance and patient commitment to treatment for BN. Perhaps it could be combined with psychotherapy in the future.

### Supplementary Information

**Additional file 1: Supplementary Fig. S1 **Forest plot of wight change. **Supplementary Fig. S2 **Forest plot of depression scores.** Supplementary Fig. S3 **Forest plot of dropouts due to adverse events.** Supplementary Fig. S4 **Forest plot of binge-eating episodes (10 weeks as the boundary).** Supplementary Fig. S5 **Forest plot of vomiting episodes (10 weeks as the boundary).

## Data Availability

The dataset supporting the conclusions of this article is included within the article (and its additional file).

## Abbreviations

BN: Bulimia nervosa
BMI: Body mass index
SMD: Standardised mean difference
WMD: Weighted mean difference
RR: Risk ratio
CI: Confidence intervals
DSM: Diagnostic and Statistical Manual of Mental Disorders
CBT: Cognitive behavioral therapy
IPT: Interpersonal psychotherapy
PROSPERO: Prospective Register of Systematic Reviews
ICD: International Classification of Diseases
BED: Binge-eating disorder
HAMD: Hamilton Depression Rating Scale
BDI: Beck Depression Inventory
SSRIs: Selective serotonin reuptake inhibitors
TCA: Tricyclic antidepressants
MAOI: Monoamine oxidase inhibitors
