# Supplementary material for: Efficacy of pharmacotherapies for bulimia nervosa: a systematic review and meta-analysis
Source: BMC Pharmacol Toxicol. 2023 Dec 2;24:72. doi: 10.1186/s40360-023-00713-7 (PMC10693702; doi:10.1186/s40360-023-00713-7)
Supplement: Supplementary file 1 — Additional file 1: Supplementary Fig. S1 Forest plot of wight change. Supplementary Fig. S2 Forest plot of depression scores. Supplementary Fig. S3 Forest plot of dropouts due to adverse events. Supplementary Fig. S4 Forest plot of binge-eating episodes (10 weeks as the boundary). Supplementary Fig. S5 Forest plot of vomiting episodes (10 weeks as the boundary). [file 40360_2023_713_MOESM1_ESM.pdf]

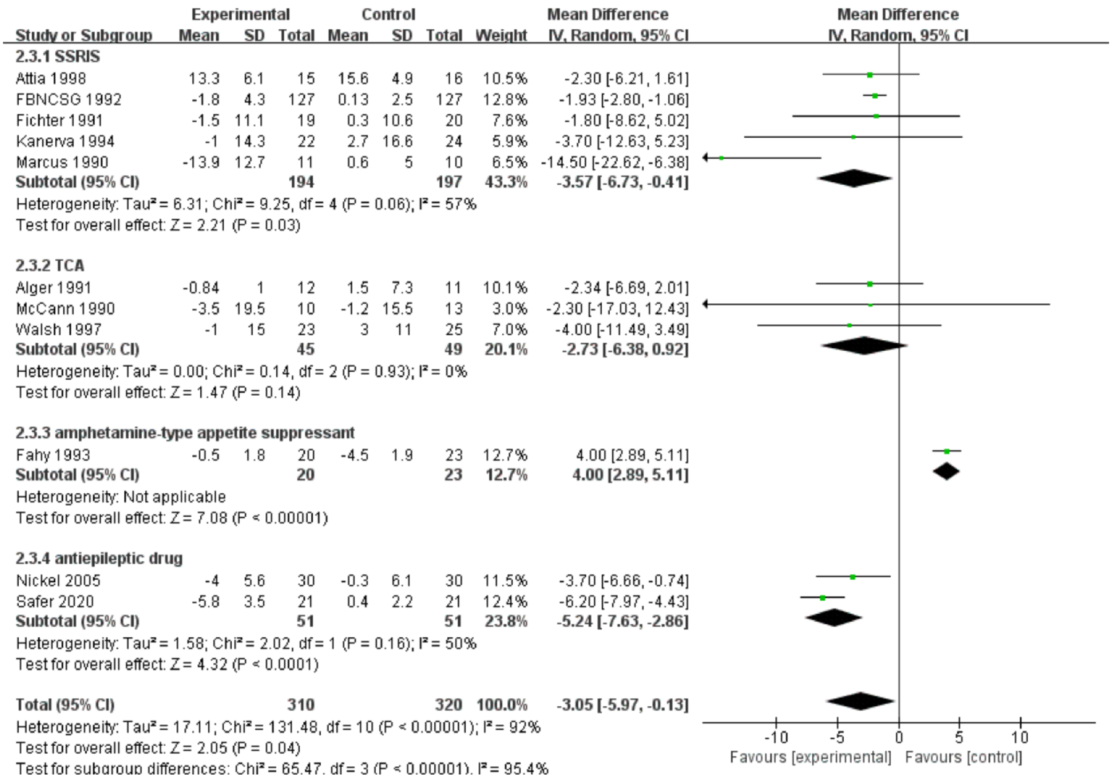

Supplementary Fig. S1 Forest plot of wight change.

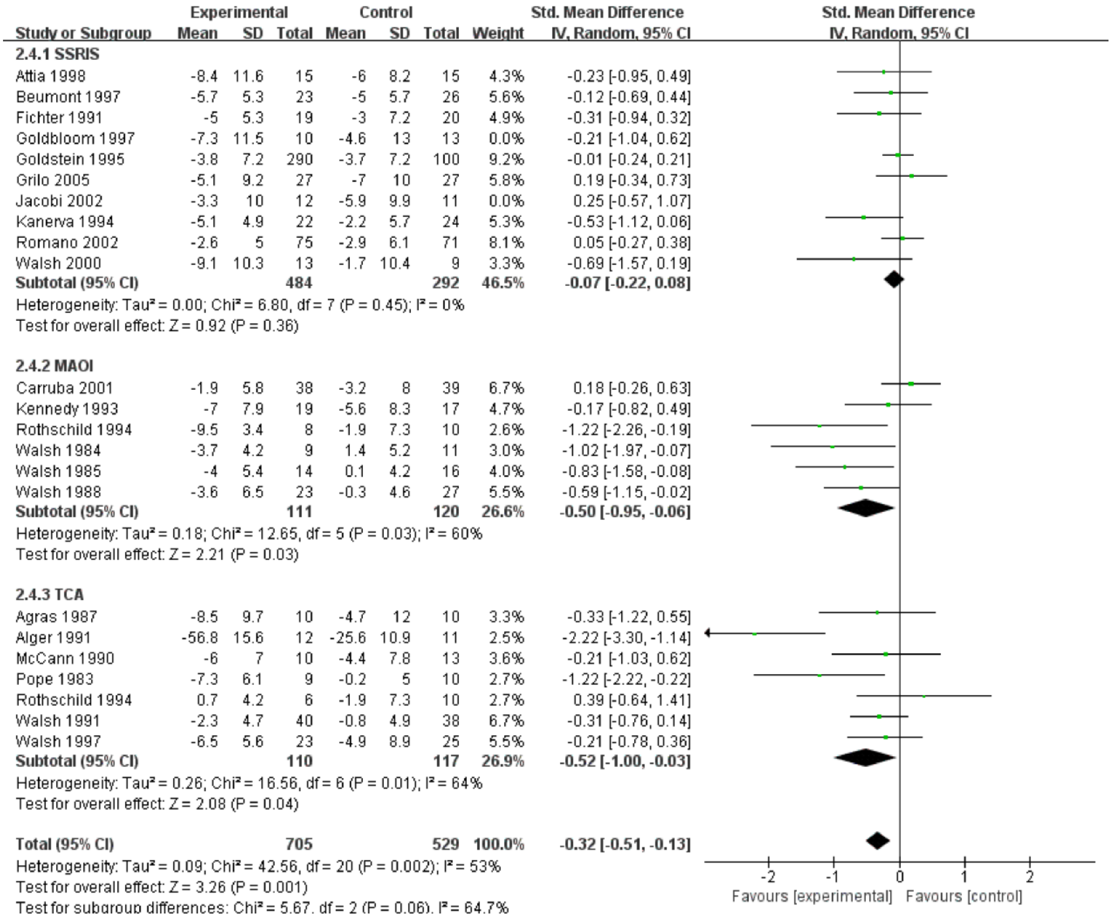

Supplementary Fig. S2 Forest plot of depression scores.

Study or Subgroup	Experimental Events	Experimental Total	Control Events	Control Total	Weight	Odds Ratio M-H, Random, 95% CI
**2.5.1 SSRIS**						
Attia 1998	4	15	4	16	6.4%	1.09 [0.22, 5.45]
FBNCSG 1992	11	129	8	129	10.6%	1.41 [0.55, 3.63]
Fichter 1997	19	37	5	35	9.1%	6.33 [2.01, 19.91]
Goldstein 1995	32	296	6	102	10.9%	1.94 [0.79, 4.78]
Grilo 2005	6	27	4	27	7.5%	1.64 [0.41, 6.64]
Kanerva 1994	0	24	1	26	2.2%	0.35 [0.01, 8.93]
Romano 2002	2	76	3	74	5.4%	0.64 [0.10, 3.94]
Wood 1993	11	296	8	102	10.6%	0.45 [0.18, 1.16]
**Subtotal (95% CI)**		**900**		**511**	**62.8%**	**1.35 [0.71, 2.58]**
Total events	85		39			
Heterogeneity: Tau² = 0.41; Chi² = 14.31, df = 7 (P = 0.05); I² = 51%						
Test for overall effect: Z = 0.91 (P = 0.36)						
**2.5.2 MAOI**						
Carruba 2001	4	38	5	39	7.5%	0.80 [0.20, 3.24]
Kennedy 1993	2	19	1	17	3.4%	1.88 [0.16, 22.83]
Walsh 1984	2	9	3	11	4.6%	0.76 [0.10, 5.96]
Walsh 1985	10	20	0	18	2.6%	37.00 [1.96, 697.35]
Walsh 1988	9	31	1	31	4.3%	12.27 [1.45, 104.10]
**Subtotal (95% CI)**		**117**		**116**	**22.4%**	**2.81 [0.64, 12.35]**
Total events	27		10			
Heterogeneity: Tau² = 1.63; Chi² = 9.76, df = 4 (P = 0.04); I² = 59%						
Test for overall effect: Z = 1.37 (P = 0.17)						
**2.5.3 TCA**						
Alger 1991	0	12	1	11	2.1%	0.28 [0.01, 7.62]
Pope 1983	2	11	1	11	3.3%	2.22 [0.17, 28.86]
Walsh 1991	5	41	0	39	2.6%	11.90 [0.64, 222.90]
**Subtotal (95% CI)**		**64**		**61**	**8.0%**	**2.18 [0.30, 16.01]**
Total events	7		2			
Heterogeneity: Tau² = 0.91; Chi² = 2.82, df = 2 (P = 0.24); I² = 29%						
Test for overall effect: Z = 0.77 (P = 0.44)						
**2.5.4 antiepileptic drug**						
Hoopes 2003	1	31	0	33	2.2%	3.30 [0.13, 83.97]
Safer 2020	2	22	2	22	4.6%	1.00 [0.13, 7.81]
**Subtotal (95% CI)**		**53**		**55**	**6.8%**	**1.41 [0.25, 7.99]**
Total events	3		2			
Heterogeneity: Tau² = 0.00; Chi² = 0.37, df = 1 (P = 0.54); I² = 0%						
Test for overall effect: Z = 0.39 (P = 0.70)						
**Total (95% CI)**		**1134**		**743**	**100.0%**	**1.61 [0.96, 2.70]**
Total events	122		53			
Heterogeneity: Tau² = 0.43; Chi² = 28.03, df = 17 (P = 0.04); I² = 39%						
Test for overall effect: Z = 1.79 (P = 0.07)						
Test for subgroup differences: Chi² = 0.92, df = 3 (P = 0.82), I² = 0%						

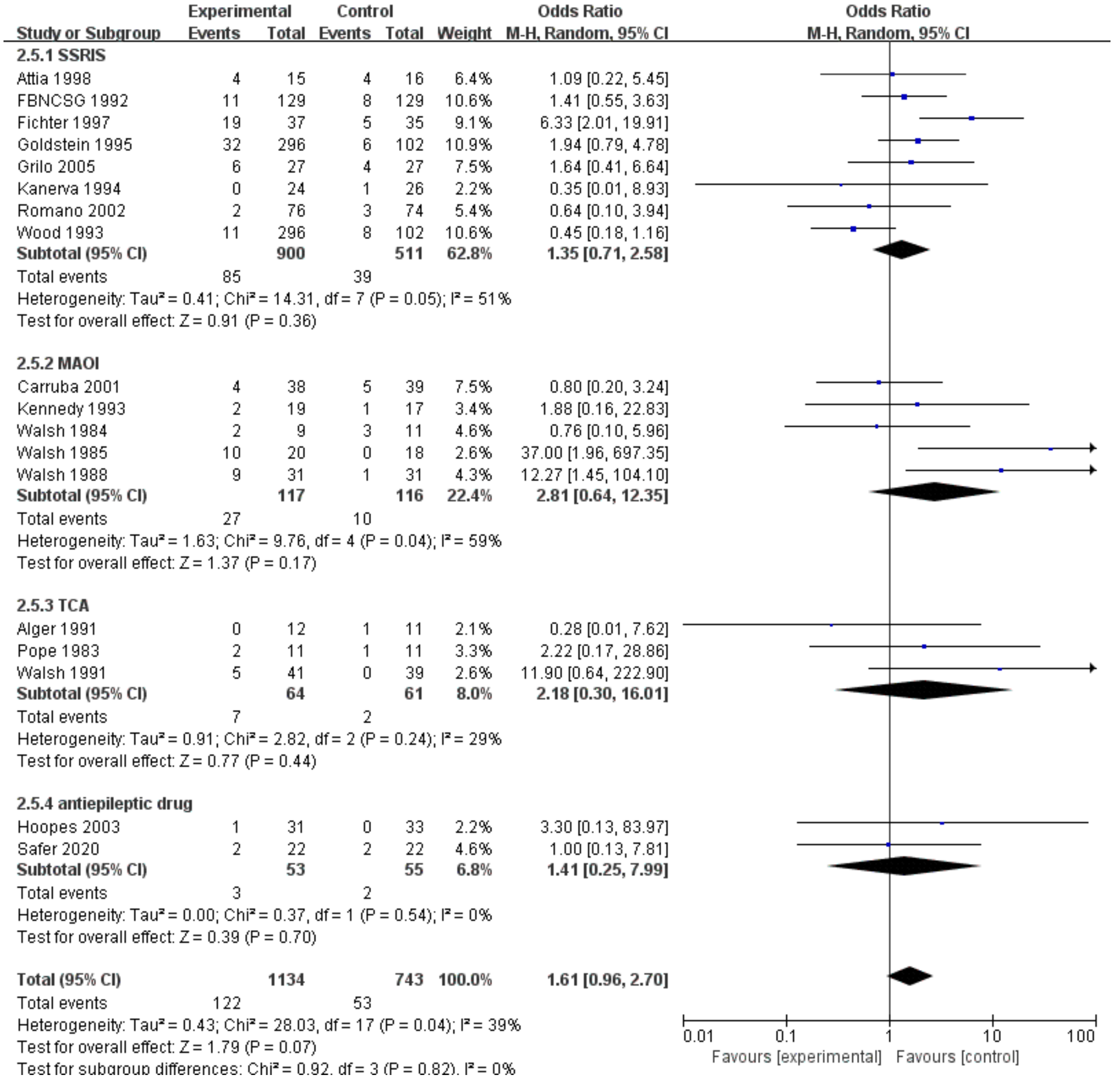

Supplementary Fig. S3 Forest plot of dropouts due to adverse events.

Study or Subgroup	Experimental Mean	Experimental SD	Experimental Total	Control Mean	Control SD	Control Total	Weight	Std. Mean Difference IV, Random, 95% CI
**3.1.1 >10 weeks**								
Agras 1987	-8.4	5.5	10	-5.6	7.9	10	3.1%	-0.39 [-1.28, 0.49]
Agras 1992	-7	5.3	12	-5.9	6.6	23	4.0%	-0.17 [-0.87, 0.53]
Goldbloom 1997	-27.8	15.1	12	-26.2	25.6	14	3.6%	-0.07 [-0.84, 0.70]
Goldstein 1995	-4.6	15.6	290	-7.5	36.6	100	6.6%	0.13 [-0.10, 0.35]
Grilo 2005	-7.6	11.7	27	-6	9.3	27	4.9%	-0.15 [-0.68, 0.39]
Jacobi 2002	-20.6	23.7	12	-12.6	26.5	11	3.4%	-0.31 [-1.13, 0.52]
McCann 1990	-2.4	2	10	0.2	3.1	13	3.2%	-0.93 [-1.81, -0.06]
Safer 2020	-16.9	15.3	22	-3.4	17.1	22	4.4%	-0.82 [-1.43, -0.20]
Sundblad 2005	-1.4	5.7	38	-2.9	6.1	29	5.2%	0.25 [-0.23, 0.74]
Walsh 1997	-6.4	4.2	23	-4.7	3.7	25	4.6%	-0.42 [-1.00, 0.15]
**Subtotal (95% CI)**			**456**			**274**	**42.9%**	**-0.21 [-0.45, 0.04]**
Heterogeneity: Tau² = 0.06; Chi² = 16.38, df = 9 (P = 0.06); I² = 45%								
Test for overall effect: Z = 1.62 (P = 0.11)								
**3.1.2 ≤ 10 weeks**								
Beumont 1997	-1.2	2.7	23	-0.8	1.7	26	4.7%	-0.18 [-0.74, 0.39]
Carruba 2001	-1.4	5.7	38	-2.9	6.1	29	5.2%	0.25 [-0.23, 0.74]
Fahy 1993	-3.5	1.3	20	-2.4	0.8	23	4.3%	-1.02 [-1.66, -0.38]
Fichter 1991	-2.63	7.9	19	-2.3	7.5	20	4.3%	-0.04 [-0.67, 0.59]
Hsu 1991	-2.9	5.1	27	-5.2	5.8	23	4.7%	0.42 [-0.15, 0.98]
Kennedy 1993	-5.6	4.9	19	-4.4	3.8	17	4.2%	-0.27 [-0.92, 0.39]
Nickel 2005	-3.4	2.7	30	-0.1	3.3	30	4.8%	-1.08 [-1.62, -0.54]
Pope 1983	-7.49	6	9	-0.18	4.6	10	2.6%	-1.32 [-2.33, -0.30]
Romano 2002	-0.5	5.9	74	0.2	6.1	71	6.1%	-0.12 [-0.44, 0.21]
Walsh 1984	-8.2	5.7	9	-0.6	6	11	2.8%	-1.24 [-2.22, -0.26]
Walsh 1985	-6.5	5.5	14	-0.6	6.2	16	3.6%	-0.98 [-1.74, -0.21]
Walsh 1988	-6.5	6.8	23	-0.8	5.4	27	4.6%	-0.92 [-1.51, -0.34]
Walsh 1991	-3.8	4.3	40	0.3	6.5	38	5.3%	-0.74 [-1.20, -0.28]
**Subtotal (95% CI)**			**345**			**341**	**57.1%**	**-0.50 [-0.80, -0.19]**
Heterogeneity: Tau² = 0.21; Chi² = 42.81, df = 12 (P < 0.0001); I² = 72%								
Test for overall effect: Z = 3.18 (P = 0.001)								
**Total (95% CI)**			**801**			**615**	**100.0%**	**-0.38 [-0.59, -0.17]**
Heterogeneity: Tau² = 0.16; Chi² = 67.17, df = 22 (P < 0.00001); I² = 67%								
Test for overall effect: Z = 3.58 (P = 0.0003)								
Test for subgroup differences: Chi² = 2.09, df = 1 (P = 0.15), I² = 52.1%								

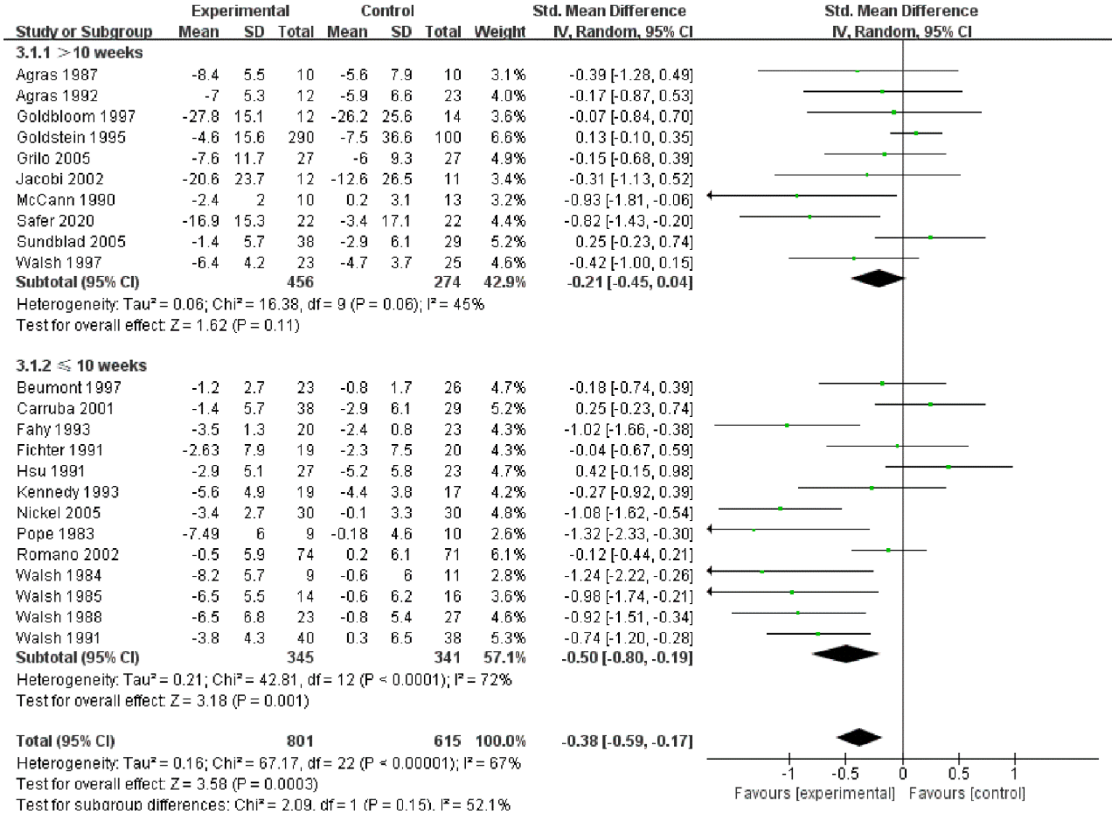

Supplementary Fig. S4 Forest plot of binge-eating episodes (10 weeks as the boundary).

Study or Subgroup	Experimental Mean	Experimental SD	Experimental Total	Control Mean	Control SD	Control Total	Weight	Std. Mean Difference IV, Random, 95% CI
**3.2.1 >10 weeks**								
Goldbloom 1997	-27.6	27.7	12	-32.8	29.8	14	3.0%	0.17 [-0.60, 0.95]
Goldstein 1995	-4.6	17.2	290	-1.6	22.6	100	34.3%	-0.16 [-0.39, 0.07]
Jacobi 2002	-15.9	27.5	12	-13.1	33.5	11	2.7%	-0.09 [-0.91, 0.73]
Walsh 1997	-25.9	12.5	23	-17.8	16.5	25	5.3%	-0.54 [-1.12, 0.04]
**Subtotal (95% CI)**			**337**			**150**	**45.3%**	**-0.18 [-0.38, 0.02]**
Heterogeneity: Tau² = 0.00; Chi² = 2.39, df = 3 (P = 0.50); I² = 0%								
Test for overall effect: Z = 1.77 (P = 0.08)								
**3.2.2 ≤10 weeks**								
Beumont 1997	-7.6	6.4	23	-5	5.6	26	5.5%	-0.43 [-1.00, 0.14]
Carruba 2001	-0.4	6.4	38	-1.5	7.9	39	8.9%	0.15 [-0.30, 0.60]
Fahy 1993	-3.3	1.5	20	-3.2	1.5	23	5.0%	-0.07 [-0.66, 0.53]
Hsu 1991	-3.9	6.1	27	-6.4	17.9	23	5.7%	0.19 [-0.37, 0.75]
Kennedy 1993	-7.6	11.7	19	-1.8	6.4	17	4.0%	-0.59 [-1.26, 0.08]
Romano 2002	-1.2	6.4	74	0.32	7.5	71	16.7%	-0.22 [-0.54, 0.11]
Walsh 1991	-3	13.6	40	0.3	17.1	38	9.0%	-0.21 [-0.66, 0.23]
**Subtotal (95% CI)**			**241**			**237**	**54.7%**	**-0.15 [-0.33, 0.03]**
Heterogeneity: Tau² = 0.00; Chi² = 6.07, df = 6 (P = 0.41); I² = 1%								
Test for overall effect: Z = 1.60 (P = 0.11)								
**Total (95% CI)**			**578**			**387**	**100.0%**	**-0.16 [-0.30, -0.03]**
Heterogeneity: Tau² = 0.00; Chi² = 8.51, df = 10 (P = 0.58); I² = 0%								
Test for overall effect: Z = 2.38 (P = 0.02)								
Test for subgroup differences: Chi² = 0.05, df = 1 (P = 0.83), I² = 0%								

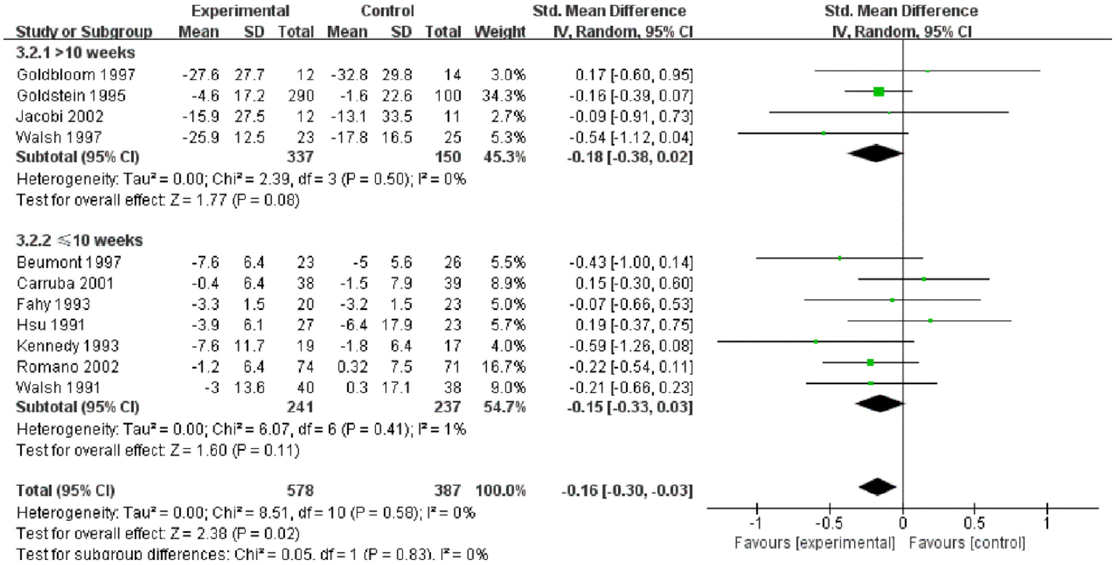

Supplementary Fig. S5 Forest plot of vomiting episodes (10 weeks as the boundary).
